# Emotion Network Analysis During COVID-19 Quarantine ‐ A Longitudinal Study

**DOI:** 10.3389/fpsyg.2020.559572

**Published:** 2020-11-10

**Authors:** Ramón Martín-Brufau, Carlos Suso-Ribera, Javier Corbalán

**Affiliations:** ^1^ Department of Acute Psychiatry Service, Román Alberca’s Hospital, Servicio Murciano de Salud, Murcia, Spain; ^2^ Department of Personality, Assessment and Psychological Treatment, Faculty of Psychology, University of Murcia, Murcia, Spain; ^3^ Departamento Psicologia Bàsica, Clínica i Psicobiologia, Faculty of Psychology, Jaume I University, Castellón de la Plana, Spain

**Keywords:** coronavirus disease 2019, network analysis, mood/emotion, pandemic impact assessment, psychopathology

## Abstract

**Introduction**: The coronavirus disease 2019 (COVID-19) emergency has imposed important challenges in the lives of individuals, particularly since the restriction of free movement. In Spain, this mandatory home confinement started on March 14, 2020. In this scenario, some calls have been made to better understand the exact impact of the quarantine on the emotional status of individuals across time.

**Materials and Methods**: On the first day that the Spanish government imposed the quarantine, our team launched an online longitudinal study to monitor emotional responses to the COVID-19 emergency over time. For 2 weeks, 187 people have responded to a daily diary on emotion functioning. An emotion network analysis was performed to study the network structure of 30 mood states and its changes during the first 2 weeks of the quarantine.

**Results**: The emotional network showed critical changes in the interactions of emotions over time. An analysis of mean emotional levels did not show statistically significant changes in mood over time. Interestingly, two different network patterns were found when the sample was divided between those with favorable responses and those with unfavorable responses.

**Discussion**: This new approach to the study of longitudinal changes of the mood state network of the population reveals different adaptation strategies reflected on the sample’s emotional network. This network approach can help identify most fragile individuals (more vulnerable to external stressors) before they develop clear and identifiable psychopathology and also help identify anti-fragile individuals (those who improve their functioning in the face of external stressors). This is one of the first studies to apply an emotional network approach to study the psychological effects of pandemics and might offer some clues to psychologists and health administrators to help people cope with and adjust to this critical situation.

## Introduction

The coronavirus disease 2019 (COVID-19) emergency imposed important challenges in the lives of individuals, particularly since the restriction of free movement and limitation of social contact started. This quarantine strategy has been used for centuries because self-isolation can help contain and control the spread of infectious diseases. However, both isolation *per se* and its uncontrollability have important negative psychological effects on individuals. Previous pandemics, such as those associated with severe acute respiratory syndrome (SARS), have been described as a mental health catastrophe due to the widespread psychopathology associated with the disease (Gardner and Moallef, 2015). In fact, some people become so anxious, distressed, avoidant, and functionally impaired under pandemics that end up requiring treatment due to the development of an emotional disorder (Wheaton et al., 2012). In this sense, although SARS was dangerous for the elderly and medically fragile, the psychological impact of SARS also inflicted a great deal of suffering in terms of the number of people affected by it and its duration (Chang et al., 2004; Washer, 2004). In another study, respondents who had been quarantined, those who worked in high-risk locations such as SARS wards, or individuals who had close friends or relatives who contracted SARS were 2–3 times more likely to have post-traumatic stress symptoms than people with lower exposure levels (Wu et al., 2009). Thus, it seems clear that mental disorders can be triggered or exacerbated by pandemic-related situational stressors (Wu et al., 2005; Gardner and Moallef, 2015; Shultz et al., 2015).

However, as evident as the effects of this quarantine during a pandemic in humans can be, we do not fully understand the psychological dynamics of mood during early quarantine stages and its longitudinal changes over the first 20 days of the COVID-19 quarantine period. Being quarantined is a complex psychological phenomenon that is hard to disentangle because there are numerous interactions between emotions and regulatory mechanisms in order to adapt to this strange and threatening new situation (Pfefferbaum and North, 2020; Suso-Ribera and Martín-Brufau, 2020). Cross-sectional studies fall short to investigate the psychological adaptation to quarantine and even pre-post studies have limitations in understanding what happens during the adaptation process (Brooks et al., 2020; Wang et al., 2020). For these reasons, longitudinal research seems to be the gold standard methodology to monitor these trajectories when attempting to better understand human psychological responses to pandemics. In addition and in contrast to the simplistic view of “one-size-fits-all” stress response to a potential traumatic situation, different trajectories have been proposed during the 2003 SARS outbreak using a latent class approach, namely, recovery, resilient, delayed, and chronic responses (Bonanno et al., 2008). To better understand these responses, a complex longitudinal analysis is needed to understand the variation and mutual influences of emotional network dynamic patterns during the early stages of the adaptation response to quarantine. This requires a new framework different to a latent approach.

Following an affective provocation, emotions interact as a dynamic and time-dependent system (Davidson, 2015). This network of emotions changes as a result of internal and external factors (Frijda, 2007). These fluctuations better characterize emotional response than mean levels of emotions (Kuppens et al., 2007; Sperry and Kwapil, 2019) and can be used to predict mood psychopathology (Wichers et al., 2015; Sperry et al., 2020). In fact, emotion dynamics may be key to understand pathways to psychopathology and well-being (Wichers et al., 2015). For these reasons, to study emotion fluctuations as a dynamic temporal network offers a good opportunity to study the response to stressful situations and increase our understanding of basic emotional responses and could suggest sooner and more successful interventions in the future.

The new field of network psychometrics has been used in recent years to investigate the complex structure of various psychiatric disorders (Fried, 2017), including depression (Fried et al., 2016), psychosis (Isvoranu et al., 2016), schizophrenia (Levine and Leucht, 2016), and anxiety (Beard et al., 2016), among others. The network perspective offers a novel way of understanding the dynamics of psychopathology (Borsboom, 2017). In contrast to viewing symptoms as reflective of underlying latent categories or dimensions, network analysis conceptualizes symptoms as constitutive of mental states, not reflective of them (McNally, 2016). At the heart of the theory lies the notion that psychopathological symptoms are causally connected through myriads of biological, psychological, and societal mechanisms. If these causal relations are sufficiently strong, symptoms can generate feedback that maintains symptomatology. In this case, the network can become stuck and develop into a disorder state (Borsboom, 2017). Ultimately, network analysis is a form of time-series analysis that has been recommended for its use in complex models where interactions between system components (e.g., different mood states) need to be modeled. This is done by graphically representing the interactions among system elements by means of edges and nodes (Gao et al., 2016). Thus, mood changes could be studied as networks, and this methodology could detect complex interactions between mood states over time that would be otherwise undetectable using pre–post methodology.

Repeated short-term assessments can detect variations in the presence and severity of states and reveal dynamic processes between them (Ebner-Priemer and Trull, 2009; Myin-Germeys et al., 2009; Bolger and Laurenceau, 2013). Network models can be used to investigate such dynamic processes in repeated assessment data from one participant [vector autoregression models (VARs)] or data from multiple participants (multilevel VAR; Epskamp et al., 2016). These models produce temporal networks depicting a directed network of the lagged associations of symptoms from one time point to the next for which Granger causal connections between symptoms are inferred (Schuurman et al., 2016). Temporal networks can then be used to identify symptoms with a high “out-strength,” that is, symptoms that are most predictive of other symptoms at the next time point (Epskamp et al., 2016).

The study of mood and its temporal evolution is important for several reasons. Moods, for example, are different to emotions in a number of characteristics, including the fact that they last longer (Ekman and Davidson, 1994). In fact, moods can have an impact on emotions (i.e., they lower the threshold that is required to trigger an emotion) (Thorndike et al., 1991). Therefore, moods can predispose individuals to experience situations in a certain manner, which can ultimately impact the way they cope with stressors (Berrocal and Extremera, 2008), such as being quarantined. Research into the determinants of mood states has been dominated by personality theories. For example, personality models like the five-factor model have shown that individuals high in neuroticism tend to present more unstable mood states (e.g., emotionality) and tend to be dominated by negative mood states (e.g., sadness and anxiety), while extraverted individuals tend to report more positive mood states (e.g., vigor; Garrity and Demick, 2001). The literature has shown, however, that mood states are influenced not only by internal factors (i.e., personality) but also by external elements (e.g., stress; Kudielka et al., 2004). As noted earlier, such changes in mood are important as they can lead to differential adaptation to adverse environments as they predispose to certain emotional states and coping efforts (Catanzaro and Mearns, 1999).

During the COVID-19 pandemic, several calls have been made to better understand the impact of the quarantine, an external stressor, on the mood status of individuals across time (Brooks et al., 2020; Lima et al., 2020). To do so, we would need to compare mood during the quarantine with mood prior to the quarantine. In the present study, however, only data after the quarantine were obtained, with the intention to explore how mood states develop over time under such strange situations using complex interaction statistical methods to study the evolution of networks of mood states under a pandemic, which can inform about human adaptation mechanisms under stressful conditions. Thus, the aim of this study was to explore the psychological dynamics of mood changes during the first stages of the COVID-19 quarantine in a sample of Spanish individuals from the general population using longitudinal data in a multilevel framework.

## Materials and Methods

### Sample and Procedure

In Spain, the mandatory home confinement officially started late on March 14, 2020. On the first day after the Spanish government imposed the quarantine, on March 15, our team launched an online longitudinal study to monitor individual adaptation to the COVID-19 emergency over time. For 2 weeks, 187 people responded to a daily diary on mood functioning.

For recruitment, a Qualtrics survey link was created and distributed during the evening of March 15 through online social networks using the virtual snowball recollection technique (i.e., asking participants to share the link with their contacts). This method was proven to be more effective than traditional snowball sampling for social sciences (Baltar and Brunet, 2012). All participants had to be older than 18 years of age, understand Spanish, and live in Spain to be eligible to participate. Eligibility was confirmed with the responses to the survey (date of birth and country of residence).

The baseline assessment was completed by 2,683 individuals (view Suso-Ribera and Martín-Brufau, 2020, for demographic information and mood state comparison of the full sample with pre-pandemic mood states). Of these, cases that missed the last 3 days of assessment and participants who missed 3 days in a row in their longitudinal assessment were excluded from the analyses. As a result, the final sample included in this longitudinal study during the first 20 days of the quarantine in Spain consisted of 187 individuals (7.4% of the baseline sample; *M*_age_ = 40.57 years, *SD* = 17.29; 78% were women; marital status = 33.13% were married, 10.07% were divorced, 56% were single, 1.2% widowed; level of education = 4.6% had a primary studies degree, 30.3% had a bachelor degree, 23.1% had a master degree, and 6.3% had a doctorate degree).

### Instruments

Sociodemographic and COVID-19-related questions included information about age, sex, marital status, income, job status, educational level, house size, number of people cohabitating, cohabitation with a child, cohabitation with pets, cohabitation with a COVID-19-infected person, perceived exposure to COVID-19, and current use of psychotropic drugs.

In a longitudinal study, we decided to study mood, as there are several differences between mood and emotion (Fox et al., 2018). Emotions are more intense, are shorter in time, are more difficult to regulate, have expressive functions, and are more influenced by specific triggers, while mood is less intense and lasts longer, so measuring mood is more suitable for our study goal (e.g., the study of a long-lasting situation on the status of individuals, as opposed to the study of specific triggers on the emotional status of individuals). The 30-item reduced version of the Profile of Mood States (POMS) Questionnaire was used to evaluate mood states (Andrade et al., 2013). This instrument was proven to be effective and robust when compared with other standardized questionnaires (Rossi and Pourtois, 2012). It evaluates six mood dimensions, namely, depression, anxiety, anger, vigor, fatigue, and friendliness. Each dimension is composed of five items with responses ranging from 0 = “Not at all” to 4 = “Extremely.” Reliability measured by Cronbach’s alpha in this study was robust for depression (alpha = 0.834), anxiety (alpha = 0.893), vigor (alpha = 0.888), fatigue (alpha = 0.871), and friendliness (alpha = 0.839).

### Statistical Analysis

A mood network analysis was performed to study the network structure of the 30 mood states and its changes during the two 2 weeks of the quarantine. Because the network framework proposes radically different views on how to understand psychological constructs and the relationship between observed variables (Bork et al., 2019), instead of trying to reduce the structure of the variables to their shared information using factor analysis, as what was done in latent variable modeling, we followed the network approach and estimated the relationship between all variables directly calculating the item interaction of mutual influences within a network structure (Borsboom and Cramer, 2013). In network analysis, the observed variables are the nodes of the network, and the estimated relations between variables are represented in the edges. Edges are not shown if their estimated value is zero. These relationships are, thus, not interpreted as relationships explained by underlying latent factors, rather, the relationships between items are interpreted as mutual causal influences between symptoms (Guyon et al., 2017).

All analyses and network graphs have been conducted with the mlVAR and the qgraph R packages, which consist of a model in which all variables in one assessment are regressed on variables of the previous assessment. This method offers a contemporaneous network and a temporal network. For brevity purposes, we only present the temporal networks in this study. Temporal networks estimate lag-1 relationships between moods after controlling for all other lagged associations (Epskamp et al., 2012, 2018). In our study, relations are interpreted as predictions of one mood over other moods at the next day controlling the influence of all other mood states. These interconnections are represented as a directed graph. Positive relationships are depicted in blue; negative relationships in red. Edges also vary in thickness depending on the strength of the connection between two moods. All connections shown are significant regressions at an alpha level of 0.05.

To study the evolution of mean levels of mood states during the first and the 20th day of the quarantine, mean differences in negative moods were compared using *t*-tests. In order to study the sample more in depth, two groups were created based on the evolution of the negative moods. We classified participants in deteriorating or improving by comparing the mood state at the beginning of quarantine with the average negative moods of the first 3 days and the last average negative moods of the last 3 days in a period of 20 days. Those who increased their negative moods were classified as deteriorating and vice versa. The group with higher negative moods (deteriorating) comprised 99 participants, and the group with lower negative moods (improving) included 89 participants.

## Results

### Total Sample Network

**Figure 1:**
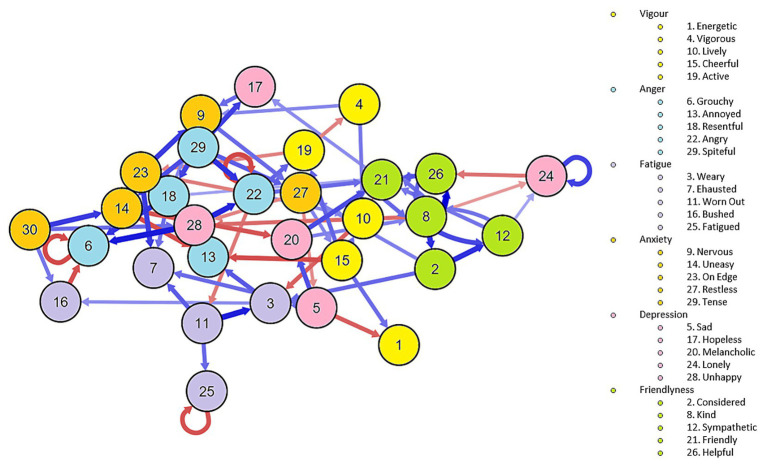
Mood network of the POMS mood dimensions.

We present the general temporal network in Figure 1. Nodes were clustered according to the corresponding dimension, with the exception of depression nodes. Nodes reflecting depression moods (in pink) were distributed near fatigue, anger, and lack of vigor in the network. “Unhappiness” (node 28) showed the strongest association both with “angry” and “grouchy.” “Sad” (node 5) activated both “melancholic” and “weary,” while “hopeless” (node 17) activated “nervous.” Nodes reflecting subjective “tension” (in orange) also showed a positive relationship between them, indicating mutual activation. For example, an increase in “on edge” (node 23) resulted in being more “nervous,” which activated feeling more “restless,” and being more “tense” activated being “uneasy.” Nodes reflecting fatigue (in light purple) were interconnected. “Worned-out” (node 11) activated subsequent “fatigue,” “exhaustion,” and “weary.” Interestingly, feeling “tense” (node 30) also increased feeling “bushed,” and feeling “on edge” increased feeling “exhausted.” Nodes reflecting anger feelings (in light blue) were also interconnected with tension and depression nodes. “Spiteful” (node 29) activated “resentful,” “angry,” “uneasy,” and “hopeless.” “Anger” nodes increased levels of “vigor” and “fatigue.”

There was a positive loop between “friendliness” items, with “kind” and “sympathetic” being the most influential nodes. There were some autoregressive feedback loops. “Fatigued,” “grouchy,” and “angry” showed negative loops with themselves, indicating an inverse relationship across days probably due to daily fluctuations in those nodes. On the other hand, “lonely” showed positive autoregressive loops, indicating an increase in loneliness during the quarantine, which is coherent with the external situation.

**Figure 2:**
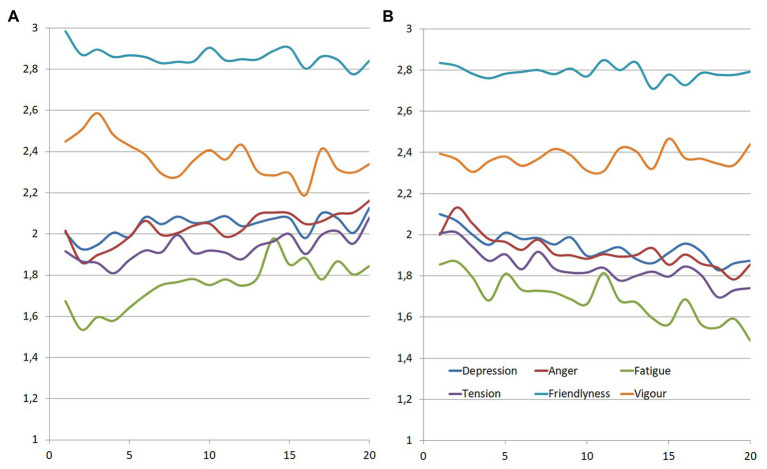
Changes in mood levels for **(A)** mood deterioration and **(B)** mood improvement.

**Figure 3:**
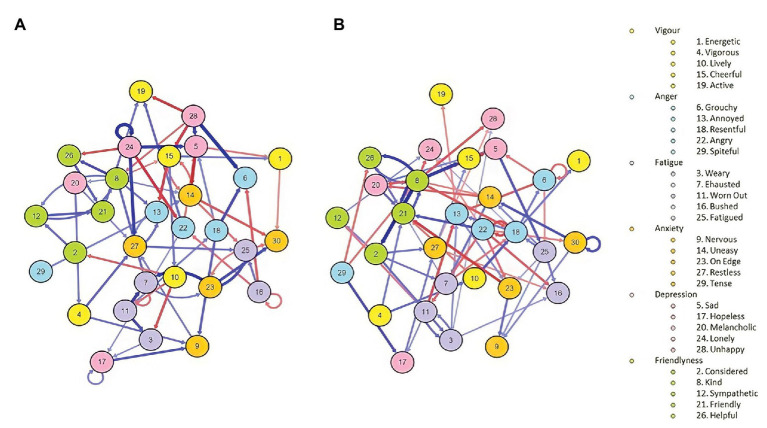
Mood networks of the POMS mood dimensions for the **(A)** deterioration and **(B)** improvement groups.

We found no statistical change in negative moods across time for the total sample when we compared averaged levels of negative moods from the first 3 and the last 3 days (*M*-day1 = 25.8; *SD* = 9.97; *M*-day 20 = 26.27; *SD* = 10.64; *t*[400] = 0.10; *p* = 0.32). Because we were interested in studying the dynamic variation of the networks, we repeated the comparison between the first and the last 3 days but divided the sample in those who increased or decreased their levels of negative affect. The evolution of mood in the two subgroups is represented in Figure 2. There was a statistically significant difference in negative moods in both the deteriorating group (*M*-day1 = 24; *SD* = 8.1; *M*-day 20 = 29.71; *SD* = 11.16; *t*[196] = −4.08; *p* < 0.001) and the improving group (*M*-day 1 = 28.66; *SD* = 11.148; *M*-day 20 = 23.37; *SD* = 9.12; *t*[176] = 3.47; *p* < 0.001). The networks for both groups are represented in Figure 3. Only temporal networks are shown in this work for clarity and brevity.

### Higher and Lower Negative Moods Networks

Figure 3 represents higher (99 participants) and lower (89 participants) negative moods at the beginning of the quarantine and its variations during the next 20 days. According to the network analysis, those who were feeling worse at the beginning of the study (graph A) showed two main nodes with a strong influence over negative moods, that is, feeling “unhappy”(node 28) and “lonely”(node 24). Feeling “unhappy” activated “sadness” and “bad temper” and inhibited “active” and “lively” moods. “Lonely” was auto-correlated, probably indicating stable tendencies toward “loneliness” moods, and activated feeling “sad” and “restless” nodes. In addition, feeling “bushed” (node 16) was positively associated with feeling “exhausted” and “weary.”

On the other hand, the network analysis revealed that those who felt better at the beginning of the quarantine (graph B) showed stronger connections between “friendliness” mood states. Feeling “kind” (node 8) was associated with other pro-social feelings. There were two negative auto-regressive nodes: feeling less “angry” and “grouchy,” perhaps as a result of a compensation mechanism to cope with the confinement. Interestingly, feeling “tense” (node 30) showed an auto-loop in the direction of indicating an increase in “tension” over days. Overall, “friendliness” mood states were inversely related to negative moods (e.g., feeling less “sad,” less “lonely,” less “melancholic,” and less “annoyed”). Note that this inverse relationship should not be interpreted as meaning that the existence of a pleasant mood results in not experiencing unpleasant moods. Both might coexist despite their negative relationship.

Overall, the network structure of those who showed higher negative moods activated depressive, anxiety, anger, and fatigue nodes, while the network structure of those who showed lower negative moods activated mood states inversely related with negative moods.

## Discussion

### Key Findings

In order to understand the complex patterns of mutual influence of mood states during the confinement, which is a potentially stressful situation, a longitudinal study was carried out during the first 20 days of the mandatory quarantine in Spain and data were analyzed with complex network techniques. This new/recent approach to the longitudinal mood change dynamics allowed us to identify different patterns of mood relationships across time. The general network showed a pattern where unhappiness, exhaustion, and anxiety influences across time were predominant, indicating an overall effortful adjustment to the lack of freedom and social distancing. To study this phenomenon more deeply, we divided the sample into negative or positive adaptations during the first 3 days compared to days 18–20 of the quarantine. With that comparison, the network structure of each group revealed distinct dynamics.

Paradoxically, individuals in which negative moods, especially loneliness and unhappiness, dominated during the first days showed an improved adaptation to the quarantine after almost 3 weeks of confinement. The reduction of exposure to stress in individuals with bad coping mechanisms might play a role in this observation. In addition to this, an overall reduction in general activation was observed. That is, while mood dynamics activated the negative valence of moods, their intensity decreased across time. In this sense, maybe isolation had this mitigating effect. This might explain why so many depressive patients search for isolation and social withdrawal as coping mechanisms (Repetti, 1992; Girard et al., 2014). On the other hand, individuals with an initial activation of positive mood states, namely, interpersonal feelings and pro-social attitudes, appeared to deteriorate with time. Although this should be interpreted with caution, it is possible that the challenges imposed by the quarantine (e.g., isolation) were initially well dealt with by optimistic and positively valenced individuals, but as isolation persisted, these individuals experienced more difficulties in maintaining their positive mood states while in social isolation.

As far as we know, only another recent study used network analysis to study COVID-19 affective responses. The authors found a direct effect of being alone and an increase in worry about COVID-19, worry about their future, and anhedonia in the temporal dynamic of the network across time (Fried, 2020). Similar to the present study, they did not find a deterioration of mental health in the students, although they did not explore whether different adaptation profiles existed in their sample, as in the present study. Several explanations could be proposed for these findings, although these are all merely hypothetical at this stage. For example, it is possible that changes could only be detected by longer assessment periods (e.g., by incorporating times of significant environmental changes, such as easing or lifting of lockdown measures). It is also possible that the stability of mental health is explained by previous mental and physical health status that is resistant to change (e.g., problems of severe fatigue or depression) or by low perceived risk (e.g., mood remains stable because perceived risk is consistently low—thanks to the quarantine). Further research is required to shed light on some of these questions, but some research has already pointed to functional fear of COVID-19 risk as an adaptive factor for public health during the current pandemic (Harper et al., 2020).

### The Depressive Network May Reflect a Temporally Convenient Adaptation Strategy

A higher tendency to negative moods seems to be the logical consequence and characterization of the loneliness and unhappiness network. Even more interesting is the resemblance between this COVID network with the bereavement network of Fried et al. (2015), where they found that the death of a spouse was strongly associated with the feeling of loneliness. This, as in the COVID network, led to unhappiness and sadness. In light of our findings, it is possible that these participants might benefit from a tendency to strategic avoidance and withdrawal from social activities. As clinical psychologists, we have seen this effect in our consultation practice at the beginning of the quarantine. That is, patients who were doing worse before the quarantine and when this started presented a better adaptation to the social isolation imposed by the quarantine. It might be the case that isolation is a known place for those with a tendency to feel such negative moods (Alpass and Neville, 2003). This is a common observation in the clinical setting, and it has been suggested that depressive patients tend to avoid this social contact in an attempt to recover from their lack of energy (Porr et al., 2010). These intertwined relationships are difficult to disentangle without the passage of time, but for some people, isolation might work for some time (e.g., during a quarantine, where social withdrawal is imposed). However, as shown by extensive literature in the field, isolation can become a problem if maintained and implemented as the main adaptation strategy to life threats (Franck et al., 2016).

There is increasing compelling evidence that links inflammatory responses with depression symptomatology (Dowlati et al., 2010). As fever and the illness behavior are characterized by quietness, reduction in motivation levels, libido, hunger, mating responses, and food search as seen in animals, we humans appear to have inherited this strategy as a starting point when there is an external threat that we cannot fight against (stay still and wait). These inflammatory-induced behaviors could be beneficial at the beginning of an infection, as they save energy and avoid threatening situations. However, this strategy, as adaptive as it can be in the long-term, can only be effectively sustained during a relatively short period of time. Maybe isolation, in parallel, serves a similar function to flight away from danger (e.g., quarantine). In this sense, it is possible that the levels of discomfort would remain approximately stable—thanks to compensation or feedback systems. These individuals would therefore maintain energy levels within adaptive ranges, which would be a characteristic of complex systems. However, this is a matter for another debate that exceeds the purpose of this study (for an evolutionary perspective of depression, see Gilbert, 2016).

### The Pro-social Network May Reflect Strategy Costs During Quarantine

On the other hand, our study revealed that the level of tension was auto-regressive in the pro-social network sample. Thus, these individuals characterized by pro-social moods, which could be considered a proxy of agreeableness, appear to regulate their moods with effortful control, inhibition of anger, and higher psychophysiological activation when using emotional suppression (Gračanin et al., 2013). Research has shown that this regulatory strategy that reduces the expression of unpleasant emotions increases the tension levels measured by psychophysiological parameters (Gross and Levenson, 1993; Srivastava et al., 2009), and it has been shown that its use may foster fresh experiences of negative affect (Pavani et al., 2017), which is perhaps one way to understand the increasing levels of unpleasant emotions experienced at the third week of the quarantine in the so-called pro-social network. The previous could also be linked to energy consumption and the increase of costly regulatory processes that might only show their deteriorating effect when other regulatory mechanisms based on social contact and kind interaction with others cannot be used anymore. This view is supported by research showing that individuals high in agreeableness recruit helpful thoughts in hostile contexts (Graziano et al., 1996), presumably in the service of controlling aggressive behavior in order to avoid rejection (Meier et al., 2008). Thus, it is possible that, although they begin from a better starting point, those using more agreeable strategies experience an increase in negative affectivity as the quarantine advances because they are more sensitive to rejection from others or social isolation.

In relation to unpleasant and pleasant emotions, it is also important to note that both might serve adaptive purposes in the face of difficult situations like the current pandemic. For example, both a pleasant mood like friendliness and an unpleasant mood like anxiety might lead to pro-social behavior and compliance with public health recommendations (Harper et al., 2020). The key point, according to recent research during the COVID-19 crisis and vast literature on the topic, lies on the tolerance and acceptance of difficult emotional states in the face of adversity (Ehrenreich et al., 2007; Suso-Ribera and Martín-Brufau, 2020). Transdiagnostic treatments, which have a focus on emotion regulation and foster tolerance to unpleasant emotions, might be important in this direction and scenarios like the present due to their applicability across emotional disorders and their feasibility in an online format (González-Robles et al., 2015).

### Limitations

An important limitation of the analysis is that we did not perform group classification based on gender or work status, presence of more people at home, history of mental health problems, and personality characteristics, to name some examples. It is possible that these groups might have experienced different levels of stress. However, there is enough longitudinal evidence showing that loneliness influences depression levels above and beyond what can be explained by initial levels of depression or demographic measures (Cacioppo et al., 2006). An alternative and plausible interpretation of these findings is that the impact of social isolation on physical and emotional health and well-being is mediated by perceived isolation rather than objective physical isolation *per se*, lack of real support, or objective demographic measures. While this is impossible to ascertain at this stage, it would highlight the important role of psychological interpretation of one’s social isolation. In other words, loneliness could be seen as a cognitive interpretation of the subjective psychological dynamic of the quarantine situation. Interestingly, these network analysis revealed a possible mechanism by which these links exist, supporting the view that psychopathology could be better understood with these techniques, as has been previously suggested (McNally, 2016). Another shortcoming refers to the generalizability of findings, which should be considered with caution due to the high attrition when comparing baseline and longitudinal data. Finally, in regard to the use of the POMS to study mood states, we acknowledge that it is possible that the dimension structure of the POMS could have influenced the clustering in the networks; future analysis should include other complementary mood states in addition to those studied by the POMS.

Despite the potential of network analysis methods, some authors have criticized their replicability problems (Forbes et al., 2017). However, these conclusions seem to be due to a misuse of the methodology both in the gathering of data phase and in network estimation (Borsboom, 2017). Thus, the estimation of networks of moods in a sample can be regarded as replicable and reliable, as shown elsewhere (Fried, 2017; David et al., 2018). Another important requirement for the consolidation of this methodology is the replication of the network in larger samples to produce more stable and robust estimates of network indices (Hevey, 2018).

### Speculations and Future Direction

Only at a speculative level, it is interesting to think about the evolution of energy and consumption of energy in a mandatory, noncontrollable situation such as a pandemic confinement. In the study, we see a reduction in overall energy, as if energy levels would decrease through the activation of predominantly inhibitory nodes of the network. One interpretation to the functioning of the network is that, as the perception of loneliness increases unhappiness, there is a progressive reduction in energy levels and an increase of despair, tension, and resentment. This could be regarded as a consequence of frustration of compensatory mechanisms to avoid psychological suffering or discomfort, which causes a threat of wasting limited energy. This hypothetical auxiliary mechanism to reduce the exhaustion of energy and resources under threat would resemble a micro-general adaptation syndrome (GAS), as Selye (1946) described it, or learned helplessness during a noncontrollable situation. According to this view, during the pandemic, something like an alarm reaction, resistance, and exhaustion phase could be described. So another possibility to explain the reduction in negative affect is that those more vulnerable to stress had already begun the adaptation process and were already in the exhaustion phase. Following this idea, it is interesting to note that the paradoxical effects could be interpreted as different timings when reacting to stressful situations. In this sense, during the SARS outbreak, different response trajectories were also clear (Bonanno et al., 2008) and resilient responders had a worse response at the beginning but recovered progressively across months. So, it is possible that those more oriented to others in the present study present a similar pattern and will be able to better adapt to the situation in the long run, but not in the mid-term (e.g., as the quarantine is maintained). These hypotheses should be tested in the future.

### Conclusion

Our study is, as far as we know, the first to identify different emotional responses in dynamic networks during the quarantine infectious outbreak. Overall, the study evidenced a stability of mood from the onset to the third week of quarantine. Interestingly, the data indicated two different trajectories characterized by differential profiles. On the one hand, those who began their confinement with an emotional profile characterized by kindness ended up experiencing a greater deterioration of mood during the quarantine. In this sense, it has been argued that possibly their expectations of social interaction as a source of well-being are more frustrated by the prolonged duration of the situation (20 days in the present study). It is possible that the lack of direct personal relationships imposes increased frustration in those who place in these pro-social emotions the key to their well-being. On the other hand, those who initiated the confinement with a greater focus on negative emotionality and awareness about the loneliness and sadness feelings associated with the COVID-19 crisis and the initial days of quarantine were more capable to adapt to their daily reality during quarantine probably because they had already anticipated the experience of loss that the quarantine entails. Despite the apparent contradiction that these data suppose with the social expectation that good health and well-being are associated with the predominance of positive emotions and pro-sociality, an important contribution of this study is that it shows that emotional dynamics are more complex than this peripheral vision of well-being, at least under unique circumstances (e.g., quarantine).

## Data Availability Statement

The datasets presented in this article are not readily available for public use. Request to access the datasets should be directed to ramonmail@gmail.com.

## Ethics Statement

The studies involving human participants were reviewed and approved by Comité ético Universidad de Murcia. The patients/participants provided their written informed consent to participate in this study.

## Author Contributions

RM-B: Data gathering, data analysis, and manuscript writing. CS-R: Data gathering and manuscript writing. JC: manuscript writing. All authors contributed to the article and approved the submitted version.

### Conflict of Interest

The authors declare that the research was conducted in the absence of any commercial or financial relationships that could be construed as a potential conflict of interest.
